# Long-Term Production and Reproductive Outcomes in Dairy Calves Following Early-Life Ultrasonographic Lung Consolidation: A Longitudinal Follow-Up Study

**DOI:** 10.3390/ani15213225

**Published:** 2025-11-06

**Authors:** Ali Sáadatnia, Gholamreza Mohammadi, Sébastien Buczinski

**Affiliations:** 1 Independent Researcher, Tehran 14155, Iran; saadat273747@gmail.com; 2 Department of Clinical Sciences, Faculty of Veterinary Medicine, Ferdowsi University of Mashhad, Mashhad P.O. Box 91773-1363, Iran; gmohamad@um.ac.ir; 3 Département des Sciences Cliniques, Faculté de Médecine Vétérinaire, Université de Montréal, St-Hyacinthe, QC J2S 8H5, Canada

**Keywords:** bovine respiratory disease, lung consolidation, thoracic ultrasonography, dairy calves, long-term outcomes, production, reproduction, culling

## Abstract

Bovine respiratory disease is a significant health concern in dairy calves, impacting short-term growth and potentially long-term productivity. While previous studies have linked early-life lung consolidation, often subclinical and diagnosed by lung ultrasonography, to reduced preweaning average daily gain, its extended effects on production and reproductive parameters remain less studied, particularly in specific geographical contexts. This study presents a follow-up analysis on a cohort of dairy calves originally monitored weekly from birth to weaning for lung consolidation via TUS in an Iranian dairy herd. Two years post-weaning, comprehensive production and reproduction data were collected and analyzed for these same animals. Our objective was to investigate the long-term associations between early-life lung consolidation and subsequent outcomes, including mature equivalent milk yield, corrected milk yield, culling risk before and during lactation, age at first breeding, age at first calving, and reproduction parameters such as services per conception and conception rate (the percentage of cows or heifers that become pregnant after a single insemination or breeding attempt). Data analysis, employing descriptive statistics, survival analysis, and non-parametric tests, revealed that while early-life lung consolidation (defined by ≥1 cm or ≥3 cm depth, and number of episodes) did not show significant associations with culling probability or mature equivalent milk yield, interesting numerical trends were observed. The study highlights the complexities of establishing clear long-term links, suggesting that while subclinical BRD can have immediate growth impacts, its chronic effects on later production and reproduction may be subtle and require larger cohorts or more targeted analysis to achieve statistical significance. The findings reinforce the need for cautious interpretation of *p*-values in the context of multiple comparisons and underscore the challenges in quantifying long-term economic consequences of early-life respiratory health.

## Simple Summary

Bovine respiratory disease is known to reduce short-term growth in dairy calves, but its longer-term effects on adult milk production and reproductive performance are less clear, particularly for subclinical pneumonia detected by thoracic ultrasonography. This longitudinal follow-up study investigated 221 female Holstein-Friesian calves from an Iranian dairy farm, whose early-life lung consolidation was identified weekly via TUS until weaning, and subsequently tracked their adult production and reproductive data for two years. Our findings revealed no statistically significant associations between early-life lung consolidation and later culling risk or mature equivalent milk yield. However, some numerical trends appeared for corrected milk yield and reproductive parameters like services per conception and conception rate. An unexpected numerical finding was that calves with consolidation tended to calve earlier. This study highlights the complexity of quantifying the precise long-term economic and biological consequences of subclinical lung consolidation, suggesting that larger, multi-farm studies are crucial to more definitively evaluate long term effects of TUS.

## 1. Introduction

Bovine respiratory disease (BRD), referring to infections of both the upper and lower respiratory tract [1,2], is one of the most prevalent and economically significant diseases affecting dairy calves worldwide [3]. In the short term, it significantly contributes to higher treatment and labor costs [4], slower growth rates [5], recurring illness [6], increased mortality [6], and the spread of pathogens [6]. The long-term effects of early-life BRD are well-established and include lower survival rates to first calving [7], reduced reproductive performance later in life [8], and decreased milk production in the future [9]. A recent systematic review and meta-analysis specifically linked calfhood respiratory disease to adverse health and performance outcomes in dairy cattle [4]. The economic impact can be substantial, with costs potentially exceeding $250 per case in young dairy heifers [10]. A major challenge in managing BRD lies in its clinical diagnosis. Traditional methods, such as visual observation of clinical signs and lung auscultation, often exhibit low sensitivity and high variability [11]. For instance, lung auscultation has been reported to have a sensitivity of less than 6% for detecting ultrasonographic lung consolidation [12], and poor diagnostic accuracy with low inter-rater reliability among veterinarians [13]. Clinical signs can be transient and difficult to identify in a significant proportion of calves, often leading to missed or misdiagnosed cases [14]. This challenge is intensified by the common presence of subclinical pneumonia, a condition in which calves develop lung lesions without showing obvious clinical symptoms [15]. Studies have reported a high incidence of subclinical pneumonia, with some indicating that up to 70.8% of calves with ultrasonographically detected pneumonia did not develop clinical signs [16,17,18]. Another factor that adds to the complexity of pneumonia is that subclinical lesions (consolidation) also have a transient nature, as highlighted by the study of Rhodes et al., 2021, which showed that some calves exhibited resolution of lesions over time, emphasizing the importance of repeated assessments to accurately diagnose and manage respiratory disease in dairy calves [12]. Thoracic ultrasonography (TUS) has therefore emerged as a promising, objective, and practical tool for on-farm BRD detection [19]. It is highly accurate, with reported sensitivities ranging from 79% to 94% and specificities from 94% to 100% for detecting BRD-related lung pathology, significantly outperforming clinical scoring systems [3,13,20]. TUS is particularly valuable for identifying subclinical lung consolidation, allowing for early detection and intervention that might otherwise be missed by clinical examination alone [5]. The detection of lung consolidation, even in the absence of clinical signs, has been consistently associated with reduced preweaning average daily gain (ADG). For example, between 21 and 50 days of age, starting from their arrival at the farm, calves with lung consolidation ≥1 cm^2^ have been shown to have a lower ADG (mean ± SE) of 0.12 ± 0.05 kg/d compared to unaffected calves [21]. The magnitude of this ADG reduction is generally around 10% to 20% of total ADG [5]. The immediate negative effects of ultrasonographic lung consolidation on preweaning growth have been established in various studies, including our previous longitudinal study conducted in an Iranian dairy herd. In that study, from birth to 8 weeks of age, by scanning once weekly, we observed that without any therapeutic intervention based on TUS diagnosis, calves with one and two or more consolidation episodes (using a ≥3 cm threshold) had significantly lower ADG compared to animals with no consolidation (0.04 kg/d reduction for one episode and 0.06 kg/d reduction for two or more episodes) [5]. Furthermore, calves with clinical pneumonia (diagnosed by the calf manager), despite receiving treatment, experienced an even larger negative effect on preweaning ADG than those with ultrasonography-diagnosed consolidation episodes alone, maybe due to more severely affected calves that were clinically detected (0.103 kg/d reduction) [5]. While the short-term impacts are clear, the long-term implications of early-life lung consolidation, particularly on subsequent production and reproductive performance, warrant further investigation using specific cohort data. Studies have linked preweaning lung consolidation to decreased first-lactation milk yield and increased risk of culling before first calving [7,9,22]. However, the specific associations between consolidation and reproductive outcomes (e.g., services per conception rate) and the varying effects of consolidation severity and duration in cohorts followed through to reproductive age remain poorly understood. Expanding on our prior longitudinal investigation, the aim of the present study was to perform a follow-up analysis on the same cohort of calves. Specifically, we aimed to investigate the long-term associations between early-life lung consolidation episodes (as identified by TUS using ≥1 cm and ≥3 cm thresholds) and subsequent production and reproductive outcomes, including mature equivalent milk yield, corrected milk yield, age at first breeding, age at first calving, services per conception, conception rate, and culling risk before and during first lactation.

## 2. Materials and Methods

### 2.1. Study Population and Original Data Collection

This study is a follow-up analysis of a cohort of female Holstein-Friesian calves previously monitored in a longitudinal study conducted in an Iranian commercial dairy farm. Detailed farm characteristics, animal management protocols, and initial data collection methodologies have been described previously [5]. Briefly, calves (*n* = 221) were monitored weekly from approximately 5–10 days of age until weaning around 8 weeks. During the initial study, thoracic ultrasonography (TUS) was performed weekly by a single trained researcher using a portable linear rectal ultrasound. The maximum depth of lung consolidation was recorded at three specific locations (right caudal to the heart, right cranial to the heart, and left caudal to the heart). Consolidation was defined using two thresholds: a more sensitive threshold of ≥1 cm and a more specific threshold of ≥3 cm. Early-life lung consolidation was either defined as presence or absence of consolidation (yes/no) and the number of weeks with consolidation further categorized in a trichotomous variable (no consolidation, 1 episode, and 2 or more episodes, respectively). Weekly body weights were recorded to calculate preweaning ADG. Other initial data collected included transfer of passive immunity (TPI) status (adequate vs. inadequate based on serum Brix %) and treatment history for clinical diseases such as BRD, diarrhea, and omphalitis, based on farmer diagnosis.

### 2.2. Production and Reproduction Outcomes

For the current follow-up study, production and reproduction data for the same cohort of calves were collected two years after the initial study was completed. This new dataset then was merged with the initial TUS findings. The production spreadsheet, contained variables such as mature equivalent milk and corrected milk, which were automatically calculated by the herd management software (Bani Asadi Livestock Management Software version 5; Bani Asadi, Iran) installed at the farm based on standard adjustment factors (e.g., age, lactation number, and season) commonly used in dairy production systems. The software utilizes monthly recorded milk yields along with animal-specific information to generate standardized production metrics, which are routinely used for herd’s performance evaluation. Reproduction parameters included age at first breeding, age at first calving, services per conception, and conception rate, culling information including pre-calving and post calving culling dates.

### 2.3. Statistical Analysis

All statistical analyses were performed using R statistical software (version 4.4.3 or 4.4.2 for certain packages) within the R studio environment. The analysis followed different steps. The production spreadsheet and TUS findings were merged based on calf identification number. The sample size for the original longitudinal study was calculated based on data from a previous study by Cramer and Ollivett (2019), which identified a 0.1 kg/day difference in average ADG between calves with and without lung consolidation [21]. With an assumed standard deviation of 0.2 kg/day, the study would require 63 calves with at least one episode of consolidation and 63 calves without consolidation, in order to detect a 0.1 kg/day difference between the two groups, with 80% power and a 95% confidence interval. To achieve this goal, 221 calves were subjected to weekly ultrasonography from birth to weaning around 8 weeks of age. Descriptive statistics obtained were used to compare various production and reproduction characteristics across the dichotomous and trichotomous consolidation categories. Continuous data were reported as median (interquartile range (IQR)). Default statistical tests (Pearson’s Chi-squared test, Fisher’s exact test, Wilcoxon rank sum test, Kruskal–Wallis rank sum test as appropriate) were used depending on the type of production outcomes and data distribution. For trichotomous outcomes, pairwise comparison was adjusted using Holm correction. For culling risk, Kaplan–Meier survival curves were generated to evaluate the probability of not being culled before calving in relation to dichotomous and trichotomous consolidation variables. An important consideration in interpreting the data is that various production and reproduction parameters (*n* = 7) were evaluated using multiple definitions of lung consolidation. This extensive testing may lead to Type I error inflation due to multiple comparisons. Therefore, care must be taken when interpreting *p*-values, particularly those between 0.01 and 0.05 divided by the number of tests (i.e., 0.007).

## 3. Results

### 3.1. Overview of Consolidation Episodes in the Cohort

The initial dataset included 221 calves. The original study was conducted on a commercial dairy farm located on the west side of Tehran, Iran. The farm housed an average of 2000 milking cows, with each cow producing an average of 11,600 kg of milk per lactation. Both male and female calves were raised under the same management and housing conditions, though only females were included in the study. All calves were weaned at approximately 8 weeks of age. The birth weights of the calves were measured using a scale with 0.1-kg accuracy (Newtech Indiamart, India, Delhi), and their weaning weights were recorded with a validated tape (ANImeter Tape, Bladel, Netherlands). The final data set consisted of 1768 TUS reports from 221 calves. In the final multivariable regression model, calves with 1 or 2 or more consolidation episodes (based on a threshold of ≥3 cm) had significantly lower Average ADG compared to those with no consolidation episode: 0.04 ± 0.02 kg/d (*p* = 0.02) for 1 episode, and 0.06 ± 0.02 kg/d (*p* = 0.002) for 2 or more episodes. When using a lower consolidation threshold (≥1 cm), there was no significant difference in ADG between calves with 1 consolidation episode and those with none (ADG reduced by 0.01 ± 0.02 kg/d, *p* = 0.60). However, calves with 2 or more consolidation episodes had a reduced ADG of 0.05 ± 0.02 kg/d (*p* = 0.003). Overall, the ADG was 0.45 ± 0.10 kg/d, and as in similar studies, calves with lesions showed approximately 10–20% decrease in ADG compared to those without lesions. The farm’s post-weaning calf rearing conditions were generally good, although no detailed data collection was performed. Heifers were not synchronized, and artificial insemination occurred at 12 to 14 months of age when they reached 385 kg in weight and 130 cm in height. Pregnancy was confirmed via ultrasound 30 days after insemination. However, a cross-sectional survey revealed that the average mature weight of the herd was 695 ± 38 kg, the average weight of one-year-old heifers was 415 ± 28 kg, and the average weight of heifers on the first day after calving was 618 ± 46 kg. Our consolidation statistics were as follows: Calves with at least one episode of consolidation with a depth ≥ 1 cm: 139 calves, representing 60%. Calves with at least one episode of consolidation with a depth ≥ 3 cm: 96 calves, representing 41%. In addition, when combined with clinical signs, 43 calves were diagnosed and treated based on clinical examination; among them, ultrasound detected consolidation ≥ 1 cm in 36 calves (84%) and consolidation ≥ 3 cm in 30 calves (70%).

**Figure 1:**
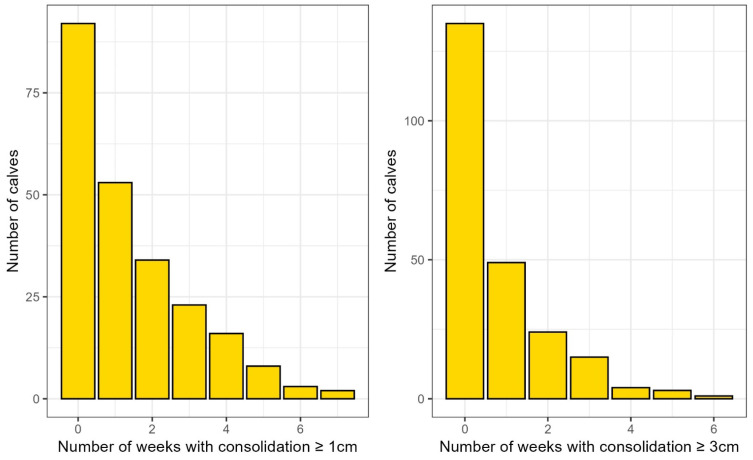
Distribution of calves based on number of weeks with consolidation ≥ 1 cm or ≥3 cm. Bar plot showing the distribution of the number of calves across the number of weeks with lung consolidation of at least 1 (**left panel**) or 3 cm depth (**right panel**).

The distribution of calves based on the number of weeks with consolidation is visualized in Figure 1.

### 3.2. Comparison of Production and Reproduction Outcomes by Consolidation Status

The main results of the outcomes conditional on lung ultrasonographic findings are indicated in Table 1. No significant association was found between the presence of consolidation ≥ 1 cm vs. consolidation < 1 cm. Surprisingly, when defining consolidation as maximal depth ≥ 3 cm, lower service per conception and higher conception rate were observed for consolidated vs. non-consolidated calves.

When considering the number of episodes of consolidation using ≥1 cm or ≥3 cm, respectively, the services per conception and the conception rate were different when defining consolidation using ≥3 cm threshold. However, post-hoc pairwise comparison using Holm correction did not find any significant pairwise difference (*p* = 0.14 for service per conception; *p* = 0.09 for conception rate). Table 2 summarizes characteristics by the number of ≥1 cm or ≥3 cm consolidation episodes (0, 1 or ≥2 episodes). No statistically significant associations were found for the parameters presented.

Overall, the herd records corresponding to the time period of our study indicated that the average age at first service among contemporaries was 13.5 ± months, with a mean first conception rate of 85 ± 9% and an average of 2.5 ± 1.6 services per conception, which are within the expected range for well-managed dairy herds.

### 3.3. Culling Risk Before Calving

**Figure 2:**
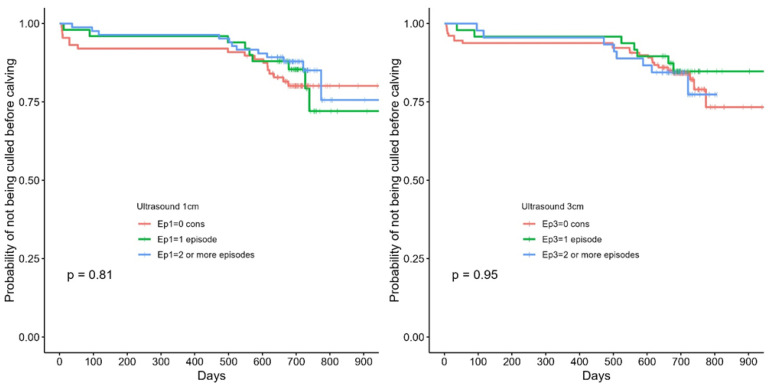
Kaplan–Meier survival curve showing the probability of not being culled before calving for calves grouped by the number of episodes (Ep) with ≥1 cm (**left panel**, Ep1) or ≥3 cm (**right panel**, Ep2) lung consolidation.

There was no effect of lung consolidation status preweaning and the risk of being culled before calving as shown in Figure 2.

## 4. Discussion

In the current follow-up study, we aimed to assess the long-term production and reproductive consequences of early-life lung consolidation in a cohort of dairy calves previously characterized by weekly thoracic ultrasonography. We did not find any adverse effect of lung consolidation in that study. While early-life lung consolidation, particularly subclinical forms, is well-established to negatively impact preweaning ADG, its direct and statistically significant long-term effects on adult production and reproduction parameters can be more challenging to elucidate, as evidenced by our findings. While this finding might seem unexpected given the emphasis on bovine respiratory disease (BRD) in the literature, it aligns with a growing recognition of the complexity and heterogeneity underlying the long-term impacts of early respiratory illness in calves. In addition, there is no information available to inform us about the spontaneous resolution of some consolidations in the postweaning period.

### 4.1. Chronological Perspective: From Calves to Lactating Cows

Chronologically, beginning with the calves, although reduced ADG is often observed in affected animals during the early stages of life, it is well-established that such animals can undergo compensatory gain. This phenomenon has been well described in feedlot cattle, where animals recovering from BRD can catch up in growth performance if appropriately managed [4,23,24]. Therefore, it is possible that any early growth delay was later compensated for masking long-term differences.

Regarding reproductive outcomes, some literature has suggested that early-life BRD can delay reproductive maturity or reduce conception rates; however, the evidence is inconsistent. For services per conception (SC) and conception rate (CR), while the number of weeks with ≥3 cm consolidation showed initial, at a glance, statistical significance (*p* < 0.05), the subsequent paired comparisons, adjusted for inflated Type I error due to multiple testing, were not significant. This is a common issue in studies with multiple comparisons and can be due to insufficient power for post-hoc tests. It implies that while there might be some underlying association, our sample size was not large enough to confirm specific pairwise differences with high confidence. However, the study by Teixeira et al. in 2017 showed that heifers with lung consolidation at weaning had a numerically lower pregnancy to first AI rate (52.5%) compared to heifers without consolidation (62.0%), with a *p*-value of 0.06 indicating a trend towards significance [22]. This suggests that lung consolidation tended to have a negative impact on conception rates, as indicated by a lower percentage of pregnancies to first AI.

Regarding age at first calving, while AFC is a widely used benchmark in both research and herd management, its interpretation requires caution, particularly when growth rate and maturity vary substantially among animals. In herds where breeding is initiated based on chronological age rather than physiological maturity, AFC can mask important variation in growth performance. In contrast, bodyweight (or bodyweight relative to mature size) is a more direct indicator of physiological maturity and metabolic readiness for reproduction. Several studies have reported that bodyweight at breeding or calving is more predictive of milk yield, fertility, and longevity than AFC alone [25,26,27,28]. However, because weighing heifers is difficult and not very practical, we followed the common approach used in similar studies and based our evaluation on AFC. Future analyses incorporating bodyweight at breeding and calving could strengthen the interpretation of how early-life health impacts lifetime productivity and reproductive efficiency. One of the more interesting, albeit unexpected findings from our study was that calves diagnosed with consolidation tended to reach calving at an earlier age (shorter age at first calving) than non-consolidated calves, although this difference was not statistically significant. Similarly, in a study by Adams and Buczinski in 2018, no significant association was found between lung ultrasonographic score and age at first calving among surviving calves [7]. Also, a study by Dunn et al. in 2018 found no significant difference in age at first calving related to lung consolidation [9]. This contrasts with some literature suggesting delayed age at first calving in calves affected by BRD. Teixeira et al. found a significantly increased age at first calving for heifers with lung consolidation (687.4 days vs. 679.8 days for healthy calves) [22]. Also, a study by Hayes et al. in 2019, from the perspective of ADG, not from the perspective of lung consolidation measurement, states that increasing ADG can have a positive impact on shortening age at first calving and lead to earlier calving [8]. It is important to acknowledge that a power calculation was not performed in our study to detect subtle differences in reproductive performance and also, such effects may be influenced by management factors post-weaning that we did not measure. For example, another potential explanation for the lack of significant differences in reproductive indices between calves with and without consolidation in our study is that our original study was observational and did not interfere with the farm’s routine treatment protocols. Consequently, consolidated calves showing clinical signs received treatment, which may have influenced key outcomes such as weight gain and production/reproductive performance. In our original study, a total of 43 calves was clinically identified and treated, of which 36 calves (84%) were identified on ultrasound with a depth cutoff ≥1 cm consolidation and 30 calves (70%) with a depth cutoff ≥ 3 cm. Therefore, adequate treatment and management of calves during or after weaning could have contributed to the lack of significant long-term detrimental effects observed in our study population. Such interventions can facilitate recovery and support subsequent growth and performance trajectories.

### 4.2. Milk Production

Our analysis indicated that mature equivalent milk yield and corrected milk yield did not show statistically significant differences across the consolidation categories, though numerical trends were observed. This is noteworthy given prior research that has established a link between early-life lung consolidation and reduced first-lactation milk production. Dunn et al. (2018) found that Holstein dairy calves with a history of lung consolidation (defined as ≥3 cm) produced 525 kg less milk in their first lactation compared to those without [9]. However, this study did not assess the calves’ average daily gain (ADG), making it unclear to what extent the observed reduction in milk yield was actually due to a decline in ADG. Similarly, a meta-analysis by Gelsinger et al. (2016) highlighted the detrimental effects of poor preweaning growth on first-lactation performance [29]. In that study, they concluded that the positive effect on lactation performance became more pronounced as the preweaning ADG increased from 0.5 kg/d to 0.9 kg/d [29]. Therefore, maintaining preweaning ADG above 0.5 kg/d can enhance first-lactation performance [29]. This may be an explanation for the lack of difference in milk production between the consolidated and non-consolidated groups in our study, as total ADG in our original study was 0.45 ± 0.10 kg/d. Our original study, based on weekly ultrasound examination of calves for 8 weeks during the pre-weaning period, reported ADG reductions in consolidated calves; a reduction of 0.04 ± 0.02 kg/d for 1 consolidation episode and a reduction of 0.06 ± 0.02 kg/d for 2 or more consolidation episodes (≥3 cm threshold), while total ADG was 0.45 ± 0.10 kg/day. The lack of statistical significance in our current study regarding milk yield, despite numerical trends, may also be attributed to the lack of power for detecting subtle differences in production parameters, a limitation explicitly acknowledged in the data analysis. The cumulative effect of chronic stress and multiple subclinical events might lead to subtle, but economically relevant, long-term losses that require larger sample sizes or more sensitive detection methods to quantify definitively. In another study by Teixeira et al. in 2017 [22], a part of their objective was to determine the association of lung consolidation detected at 60 days of life (weaning) with first lactation average weekly milk production until 90 days in milk (DIM). They found no difference in milk production between the consolidated and non-consolidated groups in the system designed in that study [22].

### 4.3. Culling Risk

Our survival analysis for culling risk before calving revealed no significant trends related to early-life lung consolidation (using either presence or absence or number of consolidation episodes with thresholds ≤ 1 cm and ≤3 cm). Similarly, Dunn et al. (2018) noted that lung consolidation did not significantly impact survival to the end of first lactation [9]. This contrasts with previous studies that identified early life lung consolidation as a predictor for culling. For example, Adams and Buczinski (2016) reported that calves with severe lung consolidation (score 4 based on 0-to-5 scoring system for lung consolidation) detected postweaning had a significantly higher risk of death or culling (26%) compared to those with lower scores (1–5%) before first lactation [7]. Also, Teixeira et al. (2017) demonstrated that heifers with lung lesions at weaning had a higher risk of being culled before calving (HR = 4.7) [22]. The lack of association between early-life (pre-weaning) lung consolidation and culling risk observed in our study may reflect biological differences between pre- and post-weaning respiratory disease. Pre-weaning BRD often arises under individual housing and milk feeding, with lesions that can resolve before breeding, whereas post-weaning BRD occurs during social mixing, dietary transition, and increased respiratory challenge, leading to more persistent pulmonary damage and growth impairment [30]. As emphasized by Bach, 2011, post-weaning health and growth are stronger predictors of reproductive performance, milk yield, and longevity than pre-weaning disease status [30]. This distinction may explain why studies focusing on post-weaning BRD (Adams & Buczinski 2016; Teixeira et al. 2017) [7,22] reported higher culling risk, while our pre-weaning cohort did not. Our findings suggest that while early-life lung consolidation can reduce preweaning growth, its long-term impact on adult production, reproduction, and culling risk may be limited, especially under proper management and treatment. This aligns with literature highlighting compensatory growth and recovery in feedlot and dairy systems, indicating that timely intervention can mitigate early setbacks [23,31]. For dairy heifer rearing, this emphasizes the importance of early detection and treatment of respiratory disease, as well as maintaining postweaning management strategies that support growth and physiological maturity. Practically, it suggests that not all calves with early subclinical lung lesions will necessarily experience long-term production losses, but vigilance in health monitoring remains critical. Our results also underscore the need for larger, multi-farm studies to clarify subtle long-term effects and to inform management practices that optimize lifetime productivity.

### 4.4. Limitations

This study was a follow-up of a cohort from a single commercial dairy farm in Iran. While this provides valuable longitudinal data, it limits the external validity and generalizability of findings to other farms or geographical areas. Postweaning management practices, including nutrition, housing, and disease prevention, were not standardized or fully documented, potentially influencing compensatory growth and long-term productivity outcomes. Other limitations included a lack of comparative herd data on culling and reproductive performance, as well as insufficient statistical power to properly evaluate reproductive outcomes. Moreover, it is probable that not all consolidation lesions are alike, and the etiological agents involved likely play a key role in determining long-term outcomes. Unfortunately, our study lacked detailed pathogen identification, which limits conclusions about specific causal agents. For example, lung consolidation due to Mycoplasmopsis bovis may have different long-term consequences compared to infections caused by Pasteurella multocida, with the latter potentially associated with lesser impacts [24]. This pathogen variability could also partly explain the heterogeneity of outcomes reported in the literature. Also, BVD virus was endemic in the original study area, potentially causing transient immunosuppression. Therefore, its direct influence on the long-term outcomes measured here is not quantifiable. Moreover, while the original study conducted weekly TUS exams, it could not rule out that some calves healed and were reinfected within the 1-week interval, or that lesions fully resolved between visits, potentially impacting the classification of some calves that were not detected with consolidation but presented transient consolidation episode of <1 week duration. Finally, sample size limitations reduced statistical power, particularly for detecting subtle differences in reproductive performance, culling risk, or first-lactation milk yield, which may have led to underestimation of the true long-term impact of early-life lung consolidation.

## 5. Conclusions

This follow-up longitudinal study on a cohort of dairy calves, previously characterized by early-life ultrasonographic lung consolidation, aimed to investigate its long-term effects on production and reproduction. These results are the illustration of the complexity of quantifying the precise long-term economic and biological consequences of subclinical lung consolidation, particularly when effects may be subtle or influenced by numerous confounding factors over an animal’s lifetime. Future research with larger sample sizes and multi-farm cohorts would be beneficial to overcome power limitations and definitively establish the long-term associations between early-life lung health and adult productivity.

## Figures and Tables

**Table 1:** Summary of Production and Reproduction Characteristics by Presence of ≥1 cm or ≥3 cm Consolidation.

Characteristic	Cons ^1^ < 1 cm	Cons ≥ 1 cm	*p*-Value ^3^	Cons < 3 cm	Cons ≥ 3 cm	*p*-Value ^3^
	N = 92 ^2^	N = 139 ^2^		N = 135 ^2^	N = 96 ^2^	
Age 1st breeding	411 (397, 428)	411 (392, 428)	0.7	412 (394, 429)	410 (391, 427)	0.5
Unknown ^4^	8	7		9	6	
Services per conception	1.50 (1.00, 2.00)	1.00 (1.00, 2.00)	0.10	2.00 (1.00, 2.00)	1.00 (1.00, 2.00)	0.005
Unknown	8	7		9	6	
Conception rate	0.50 (0.50, 1.00)	1.00 (0.50, 1.00)	0.13	0.50 (0.50, 1.00)	1.00 (0.50, 1.00)	0.008
Unknown	8	7		9	6	
Culled pre-calving	17 (18%)	23 (17%)	0.7	24 (18%)	16 (17%)	0.8
Age at 1st calving (month)	23.5 (22.7, 24.4)	23.4 (22.6, 24.4)	0.5	23.5 (22.8, 24.5)	23.2 (22.6, 24.1)	0.071
Unknown	21	27		30	18	
Culled during lactation	1 (1.3%)	2 (1.7%)	>0.9	1 (0.9%)	2 (2.5%)	0.6
Unknown	17	23		24	16	
Mature equivalent milk (kg)	13,365 (9307, 15,037)	12,023 (9635, 15,193)	0.8	13,727 (9502, 15,495)	11,421 (9590, 14,742)	0.4
Unknown	19	27		26	20	
Corrected milk (kg)	10,801 (10,025, 11,654)	11,142 (10,546, 11,925)	0.065	10,896 (10,093, 11,682)	11,011 (10,566, 11,925)	0.3
Unknown	19	27		26	20	

Characteristics of study calves stratified by the presence or absence of at least one episode of lung consolidation ≥ 1 cm (left panel) or ≥3 cm (right panel). Values are presented as n (%) for categorical variables and median (IQR) for continuous variables. ^1^ Cons: Consolidation. ^2^ Median (Q1, Q3); n (%). ^3^ Wilcoxon rank sum test; Pearson’s Chi-squared test; Fisher’s exact test. ^4^ Unknown values represent instances where the data for those specific fields is missing.

**Table 2:** Summary of Production and Reproduction Characteristics by Number of episodes with ≥1 cm or ≥3 cm Consolidation.

Characteristic	0 Cons ≥ 1 cm	1 Episode ≥ 1 cm	2 or More Episodes ≥ 1 cm	*p*-Value ^2^	0 Cons ≥ 3 cm	1 Episode ≥ 3 cm	2 or More Episodes ≥ 3 cm	*p*-Value ^2^
	N = 92 ^1^	N = 53 ^1^	N = 86 ^1^		N = 135 ^1^	N = 49 ^1^	N = 47 ^1^	
Age 1st breeding	411 (397, 428)	414 (396, 428)	409 (388, 428)	0.8	412 (394, 429)	418 (400, 431)	405 (383, 422)	0.068
Unknown ^3^	8	2	5		9	3	3	
Services per conception	1.50 (1.00, 2.00)	1.00 (1.00, 2.00)	1.00 (1.00, 2.00)	0.2	2.00 (1.00, 2.00)	1.00 (1.00, 2.00)	1.00 (1.00, 2.00)	0.018
Unknown	8	2	5		9	3	3	
Conception rate	0.50 (0.50, 1.00)	1.00 (0.50, 1.00)	1.00 (0.50, 1.00)	0.2	0.50 (0.50, 1.00)	1.00 (0.50, 1.00)	1.00 (0.50, 1.00)	0.026
Unknown	8	2	5		9	3	3	
Culled pre-calving	17 (18%)	10 (19%)	13 (15%)	0.8	24 (18%)	8 (16%)	8 (17%)	>0.9
Age at 1st calving (month)	23.5 (22.7, 24.4)	23.3 (22.6, 24.4)	23.4 (22.6, 24.4)	0.8	23.5 (22.8, 24.5)	23.2 (22.7, 24.3)	23.1 (22.3, 24)	0.14
Unknown	21	12	15		30	8	10	
Culled during lactation	1 (1.3%)	1 (2.3%)	1 (1.4%)	>0.9	1 (0.9%)	2 (4.9%)	0 (0%)	0.2
Unknown	17	10	13		24	8	8	
Mature equivalent milk (kg)	13,365 (9307, 15,037)	11,706 (9564, 15,583)	12,173 (9796, 14,899)	>0.9	13,727 (9502, 15,495)	11,899 (9615, 14,860)	10,693 (9497, 14,655)	0.6
Unknown	19	12	15		26	12	8	
Corrected milk (kg)	10,801 (10,025, 11,654)	11,043 (10,366, 12,049)	11,195 (10,565, 11,879)	0.2	10,896 (10,093, 11,682)	11,043 (10,697, 12,049)	10,854 (10,422, 11,879)	0.2
Unknown	19	12	15		26	12	8	

Characteristics of study calves stratified by the number of weekly examinations with ≥1 cm or ≥3 cm consolidations. Values are presented as n (%) for categorical variables and median (IQR) for continuous variables. *p*-values derived from Pearson’s Chi-squared test, Fisher’s exact test, or Kruskal–Wallis rank sum test. ^1^ Median (Q1, Q3); n (%). ^2^ Kruskal–Wallis rank sum test; Pearson’s Chi-squared test; Fisher’s exact test. ^3^ Unknown values represent instances where the data for those specific fields is missing.

## Data Availability

The data used in this study consist of production records collected from dairy cows over a two-year period. Due to privacy and ethical restrictions, these data are not publicly available but can be obtained from the corresponding author upon reasonable request.
